# Feasibility and preliminary effectiveness of a psychosocial support program for adolescent and young adult cancer patients in clinical practice: a retrospective observational study

**DOI:** 10.1007/s00520-023-07596-8

**Published:** 2023-02-02

**Authors:** Takatoshi Hirayama, Maiko Fujimori, Yoshinori Ito, Yuji Ishida, Akemi Tsumura, Miwa Ozawa, Naoko Maeda, Kazuhito Yamamoto, Sakie Takita, Makiko Mori, Kyoko Tanaka, Keizo Horibe, Tatsuo Akechi

**Affiliations:** 1 Department of Psycho-Oncology, National Cancer Center Hospital, Tokyo, Japan; 2 Division of Supportive Care, Survivorship and Translational Research, National Cancer Center Institute for Cancer Control, 5-1-1 Tsukiji, Chuo-Ku, Tokyo, 104-0045 Japan; 3 Department of Psychiatry and Cognitive-Behavioral Medicine, Nagoya City University Graduate School of Medical Sciences, Nagoya, Japan; 4 Department of Pediatrics, Shizuoka Cancer Center, Shizuoka, Japan; 5 Authorized Non-Profit Organization, Yokohama Children’s Hospice Project, Yokohama, Japan; 6 Department of Pediatrics, St. Luke’s International Hospital, Tokyo, Japan; 7 Department of Pediatrics, National Hospital Organization Nagoya Medical Center, Nagoya, Japan; 8 Department of Hematology and Cell Therapy, Aichi Cancer Center, Nagoya, Japan; 9 Department of Nursing, Sugiyama Jogakuen University, Nagoya, Japan; 10 Department of Hematology/Oncology, Saitama Children’s Medical Center, Saitama, Japan; 11 Division of Pediatric Consultation Liaison, Department of Psychosocial Medicine, National Center for Child Health and Development, Tokyo, Japan; 12 Clinical Research Center, National Hospital Organization Nagoya Medical Center, Nagoya, Japan

**Keywords:** Adolescent and young adults, Needs assessment, Program evaluation, Psychosocial support, Screening, RE-AIM

## Abstract

**Purpose:**

Adolescent and young adult cancer patients (AYAs) often experience profound psychological distress, with various unmet supportive care needs that can be alleviated with appropriate screening and attention by healthcare workers. The Distress Thermometer and Problem List-Japanese version (DTPL-J) is our previously developed screening tool to facilitate individual support of AYAs. This study evaluated the feasibility and preliminary effectiveness of a psychosocial support program based on the DTPL-J for AYAs in clinical practice.

**Methods:**

This multicenter, retrospective, observational study included 19 of 126 wards and 9 of 75 outpatient clinics at 8 institutions in Japan. Over 200 patients were expected to participate during the eligibility period. Patients participated in a support program at least once, and approximately once a month based on the DTPL-J results. The program was evaluated using the RE-AIM (Reach, Effectiveness, Adoption, Implementation, and Maintenance) implementation framework.

**Results:**

The screening rate of the 361 participants was 90.3%, suggesting high feasibility. Distress Thermometer scores, the number of supportive care needs, and the rates of AYAs with high distress were significantly reduced 1 month after screening (*p* < 0.05), suggesting the preliminary effectiveness of the program. The program was continued at the 8 institutions as part of routine care after the study.

**Conclusion:**

Analysis using the RE-AIM suggested the sufficient feasibility and preliminary effectiveness of a psychosocial support program based on the DTPL-J for AYAs.

**Trial registration:**

University Hospital Medical Information Network (UMIN CTR) UMIN000042857. Registered 25 December 2020—Retrospectively registered.

## Introduction 

Adolescent and young adult cancer patients (AYAs) are defined as those aged 15 to 39 at the time of initial cancer diagnosis [1]. Approximately 87,000 and 20,000 AYAs in the USA and Japan, respectively, are newly diagnosed each year, corresponding to approximately 4.5% and 2.3% of all people diagnosed with cancer [2, 3].

AYAs require more support than older adults or younger children with cancer [4]. Though they have age-specific various needs [5–12], many of these needs are unmet [11, 13]. In a previous study, more than 70% of AYAs reported unmet supportive care needs [14], which have been found to lead to psychological distress [15] and deterioration of quality of life [16]. It has been reported that about 60% of AYAs experience psychological distress [17]. Therefore, to address these unmet needs and improve psychological distress and quality of life, psychosocial care as well as medical care for AYAs is needed. Screening for distress and unmet needs were reported to lead to better support provision and guidance on service development for AYAs [18]. In this study, we defined “psychosocial care” as comprehensive care not only for medical care but also for various psychological and social unmet needs.

In Japan, the Ministry of Health, Labour and Welfare released the Third Basic Plan to Promote Cancer Control Programs in 2018 [19]. This policy aims to enhance cancer control in the adolescent and young adult (AYA) population and promote the construction of a network of AYA support teams. Although the importance of supporting AYAs is recognized among healthcare workers, the number of AYAs at each hospital is small and the primary cancer site varies [20]. Thus, it is difficult for healthcare workers to gain experience in providing medical care and support to this population.

The Distress Thermometer and Problem List (DTPL), which was developed by the National Comprehensive Cancer Network, was found to be useful in Singapore for identifying clinically significant psychological distress in AYAs [21]. In Australia, an AYA-specific screening tool based on the DTPL helped healthcare workers support psychosocial coping in AYAs [22]. The tool was validated in a multinational study conducted primarily in English-speaking countries [18]. Psychosocial support using the tool is being implemented as a national project in Australia [23]. To assess distress and supportive care needs in AYAs, the DTPL-J for AYAs was developed by our group as a screening tool based on the DTPL [24], and the feasibility, validity, and reliability of the tool were suggested [25]. A psychosocial support program including the DTPL-J for AYAs may allow healthcare workers to support the needs of AYAs beginning soon after their diagnosis.

This program designed to provide psychosocial support to AYAs in cooperation with multidisciplinary experts was developed with reference to the support system of the NCCH [26]. In this program, healthcare workers provided screening using the DTPL-J for AYAs, identified the needs of AYAs, and determined the necessary support. Since the previous study was feasible at a single cancer center [25], the feasibility and preliminary effectiveness of this program were examined using the Reach, Effectiveness, Adoption, Implementation, and Maintenance (RE-AIM) framework at multiple institutions with a view to clinical implementation.

In recent years, the RE-AIM framework has been used to evaluate the feasibility and preliminary effectiveness of support programs in various fields [27–29]. This framework organizes aspects related to the implementation and outcomes of intervention programs into five categories: Reach, Effectiveness, Adoption, Implementation, and Maintenance [30]. The RE-AIM aims to assess the impact (external validity or generalizability) of public health intervention programs under complex real-world conditions [31].

This multicenter study aimed to evaluate the feasibility and preliminary effectiveness of a psychosocial support program based on the DTPL-J for AYAs, using the 5 dimensions of the RE-AIM framework.

## Methods

### Study design

We conducted a multicenter, retrospective, observational study to evaluate the feasibility and preliminary effectiveness of a psychosocial support program for AYAs. This study was approved by the National Cancer Center Institutional Review Board (approval number, 2020–071) and was conducted in accordance with the principles of the Declaration of Helsinki. The requirement for informed consent was waived due to the retrospective design, and opt-out information was published on the website of the National Cancer Center Hospital (NCCH) in Japan. This study was registered at the UMIN Clinical Trials Registry (UMIN000042857).

The following 8 institutions participated, all of which were members of a research group defined by a Grant-in-Aid from the Japanese Ministry of Health, Labour and Welfare: NCCH, Nagoya City University Hospital (NCUH), Shizuoka Cancer Center (SCC), St. Luke’s International Hospital, Nagoya Medical Center (NMC), Aichi Cancer Center (ACC), Saitama Children’s Medical Center (SCMC), and the National Center for Child Health and Development (NCCHD).

The sample size was pre-set to 200 patients (NCCH, 100; NCUH, 10; SCC, 20; St. Luke’s International Hospital, 20; NMC, 10; ACC, 20; SCMS, 10; and NCCHD, 10), based on the average numbers of patients at the targeted departments of the 8 institutions over the previous 3 years, and under the assumption that about 20% of the patients would drop out. Although the rate of dropout was approximately 10% in our previous study at the NCCH [25], the value of dropout (20%) in this multicenter study was set higher because the NCCH support system for AYAs was more robust than at other institutions [26]. NCCH is a comprehensive cancer center, with rich resources of multidisciplinary experts as well as a clinical trial infrastructure. In a previous study using DT, about 20% of the AYAs dropped out [32].

### Psychosocial support program for AYAs

A program designed to provide psychosocial support to AYAs in cooperation with multidisciplinary experts was developed with reference to the support system of the NCCH [26]. Based on the results of a retrospective analysis of the system, an expert panel was convened, consisting of 5 oncologists, 2 psycho-oncologists, 2 psychologists, 2 pediatricians, 2 palliative care physicians, 2 nurses, 1 patient advocate, and 4 education specialists. This panel examined the external validity of the developed support program, summarized the common program elements and those unique to each institution, and created an implementation manual for a support program that could be established given the resources of each institution. A manual was developed in advance, and the common practice was to conduct screening as a core component, followed by assessment by nurses as primary care, and referral to secondary care as needed.

Because each institution had different multidisciplinary expert resources, the secondary care to which referrals were made for each need was determined in advance. The manual included the role of each expert and how they should support AYAs in order to ensure the professionalism of psychosocial support.

In this program, healthcare workers provided screening using the DTPL-J for AYAs, identified the needs of AYAs, and determined the necessary support. The DTPL-J for AYAs consisted of the Distress Thermometer (DT) and a list of 49 problems potentially experienced by patients [25]. A nurse performed screening as soon as possible after inpatient admission, and at the first visit in the case of outpatients. The AYAs participated in the program at least once, and some departments also conducted the program approximately once a month based on the DTPL-J results.

Primary support was handled by nurses. After screening to determine patients’ needs, nurses provided information on necessary support. In addition, with the consent of the patients, the obtained information was shared with the primary care team. The consent was verbally obtained from AYAs in the course of a treatment interaction. If not an adult, verbal consent was obtained not only from them, but also from their parents/guardians. This procedure was approved by prior ethical review. Patients who required intervention received expert support at an early stage. The main occupations of the experts involved in secondary support were attending physicians, specialized nurses, psychologists, and medical social workers.

Attending physicians were responsible for incorporating information such as the physical, psychological, and social backgrounds of the patients into their treatment plan. They shared information with multidisciplinary experts at conferences and considered the best medical care. Specialized nurses played a central role in sharing information and collaborating with multidisciplinary experts so that healthcare workers could provide comprehensive support to AYAs and their families. In addition to administering direct care such as daily primary care, therapeutic decision support, and self-care support, specialized nurses also educated and provided logistics support to nurses responsible for primary support. Psychologists addressed psychological distress that could not be alleviated by primary support. They provided psychological care not only to AYAs and their families, but also to healthcare workers who supported and facilitated the relationship between AYAs, their families, and healthcare workers. Medical social workers played roles such as presenting information on financial issues and the use of social systems, providing support regarding employment and school attendance, and modifying the medical treatment environment.

In addition to the above, it was desirable that various additional multidisciplinary experts pharmacists, nutritionists, rehabilitation staffs, psychiatrists, child life specialists, appearance care staff, child support, and palliative care team be involved in the program according to the resources of each institution.

### Data sources

The relevant data sources for each of our study aims, including how they map onto the RE-AIM framework, are listed in Table 1.

**Table 1:** Aims, data sources, and analysis mapped to the RE-AIM framework

Aims: to describe and evaluate	Relevant dimensions of the RE-AIM framework	Data sources	Analysis
Setup of the psychosocial support program for adolescent and young adult cancer patients (AYAs)	Adoption: the absolute number, proportion, and representativeness of settings and intervention agents who are willing to initiate a program	Interviews with healthcare professionals	Milestones in the setup of the psychosocial support program for AYAs - Reported factors facilitating set-up - Reported factors hindering set-up
Patients who participated in the psychosocial support program for AYAs	Reach: The absolute number, proportion, and representativeness of individuals who are willing to participate in a given initiative	Routine clinical data	Descriptive statistics were used to retrospectively calculate the number of cases at each institution, the types of specialists involved in each patient's care, and the percentage of items on the problem list that were checked by each patient
The extent to which the psychosocial support program for AYAs was implemented as intended	Implementation: The intervention agents’ fidelity to the various elements of an intervention’s protocol	Routine clinical data Interviews with healthcare professionals	The percentage of participants who underwent the first screening and reasons for failure to conduct screening.
How well the psychosocial support program for AYAs worked	Effectiveness: The impact of an intervention on important outcomes, including potential negative effects, quality of life, and economic outcomes	Routine clinical data	To evaluate program efficacy, a second screening was performed if clinically feasible. For the first and second screenings, the DT score and the number of checked items were compared using the t-test.
The sustainability of the psychosocial support program for AYAs as part of routine care	Maintenance: the extent to which a program or policy becomes institutionalized or part of routine organizational practice or policies	Interviews with healthcare professionals	Milestones in the sustainability of the psychosocial support program for AYAs - Reported factors facilitating sustainability - Reported factors hindering sustainability

### Routine clinical data

Beginning in August 2020, each institution implemented the program for AYAs in clinical practice. The eligibility period was from August 2020 to March 2021. After the end of the eligibility period on April 1, 2021, collaborators from each institution extracted the data listed in Table 1 to be analyzed. These data were linked to the research registration numbers in the medical records, and databases were created for analysis. The databases were collected and evaluated by the research office of the NCCH. The data manager (T. H.) of the research office of the NCCH checked the accuracy of the data. If any conflict, errors, and missing data were found in the databases, the data manager (T.H.) discussed them with the collaborator in charge of each facility to resolve the problem.

The data extracted from the medical records and DTPL-J for AYAs included the following: age, gender, cancer type (primary site), stage, days from diagnosis, treatment setting, treatment type (outpatient or inpatient), DT score, checked items on the problem list, and experts who supported each patient’s needs.

Regarding the items on the problem list, St. Luke’s International Hospital and NMC added several items such as marriage, caregiving, heredity, and available systems and services to the DTPL-J for AYAs.

### Interviews

Before the program was implemented in clinical practice, healthcare professionals at the 8 institutions were interviewed about factors that facilitated or hindered program setup. After the end of the eligibility period, the same individuals were interviewed about the benefits and challenges involved in implementing the program and whether or not the program was continued after the eligibility period. One interviewer (T. H.) conducted a total of two interviews per institution online according to the manual. Each interview lasted 1 h with at least one member of the research team in each institution. The interviews were recorded and the conversations were transcribed by one researcher (T. M.). As interview participants, M. F., Y. I., Y. I., A. T., M. O., N. M., K. Y., S. T., M. M., K. T., K. H., and T. A. participated in two interviews in their institution. The interview content was classified based on similarities and differences between elements that promoted or impeded program sustainability. Content analysis of the interview data was conducted by a psychiatrist (T. H.) and a psychologist (M. F.) using the KJ method [33].

### Statistical analysis

We mapped our study aims, specifically the feasibility and preliminary effectiveness of the program, to the (re-ordered) RE-AIM dimensions presented below.

To assess feasibility, we described each of the following: the establishment (Adoption) of the program, including factors that facilitated or hindered setup; described and evaluated the extent to which patients participated in the program (Reach); assessed the extent to which the program was delivered as intended (Implementation), along with relevant facilitating and obstructing factors; and the sustainability of the program as part of routine care (Maintenance).

To assess preliminary effectiveness, we evaluated how well the program worked (Effectiveness).

Based on our previous study [25], we pre-determined that the program was feasible if a participation rate (Reach) of ≥ 90% was achieved. This rate was defined as the number of subjects who completed the first screening divided by the total number of patients who participated in the program, multiplied by 100. Although the program participation rate was 91.6% at the NCCH [25], the reference value (90%) was set slightly lower because the NCCH support system for AYAs was more robust than at other institutions [26].

Descriptive statistics were used to retrospectively calculate the number of cases at each institution, the types of specialists involved in each patient’s care, and the percentage of items on the problem list that were checked by each patient.

If clinically feasible, the utility of the program was evaluated by conducting a second screening about 1 month after the first, or at the time of discharge if the inpatient was discharged within 1 month.

The DT score and the number of checked items at the first and second screenings were compared using the *t*-test. The overall patient sample, including individuals who missed the second screening, was analyzed using the unpaired-samples *t*-test. Only patients who completed both the first and second screenings were analyzed using the paired-samples *t*-test. A 2-sided *p* value < 0.05 was used to indicate statistical significance. A DT score of ≥ 5 was defined as high distress because a score of 5 resulted in the best clinical screening cutoff on DT in AYAs [18]. The rates of AYAs with high distress (the number of patients with a DT score of ≥ 5 points)/(the number of patients who reported any DT score) before and after receiving the support program were compared using the chi-square test.

## Results

### Program setup (Adoption)

Nineteen of 126 wards (15.1%) and 9 of 75 (12%) outpatient clinics at 8 institutions participated. Factors that facilitated setup were as follows: (a) the impetus for building a system to support AYAs (awareness of the need for AYA support and clarification of the procedure of this support); (b) increased opportunities to share information with multidisciplinary experts to help them support AYAs (study meetings and periodic conferences); and (c) provision of early and appropriate support (establishment of a screening system and clarification of consultation services). Reported barriers to setup were as follows: (a) concerns about an increased daily burden in clinical practice; (b) burden on patients in terms of responding to screening; and (c) gaining the approval of the targeted departments to implement the program and establishing a system of cooperation.

### Program participants (Reach)

A total of 361 of 1262 (28.6%) targeted patients participated in the study. The number of patients who participated at each institution was as follows: 138 of 202 (68.3%) at the NCCH, 86 of 86 (100%) at the SCC, 19 of 219 (8.7%) at the ACC, 50 of 50 (100%) at the NCUH, 19 of 115 (16.5%) at the NMC, 31 of 495 (6.3%) at St. Luke’s International Hospital, 11 of 32 (34.4%) at the SCMC, and 7 of 63 (11.1%) at the NCCHD.

Regarding participants’ demographic characteristics, 206 males (57.1%) and 155 females (42.9%) with an average age of 28.2 years were included. Three hundred four subjects (84.2%) were inpatients. Cancer types were bone and soft tissue cancer (*n* = 131, 36.3%), breast cancer (*n* = 39, 10.8%), gynecological cancer (*n* = 35, 9.7%), hematological cancer (*n* = 34, 9.4%), brain tumor (*n* = 26, 7.2%), and others (*n* = 96, 26.6%). Stage IV (*n* = 91, 25.2%) was the most common cancer stage at diagnosis, followed by recurrence (*n* = 45, 12.5%). The most common treatment setting was curative (*n* = 193, 53.5%).

Patient problems and the experts who supported patient needs are shown in Table 2. The most common occupations of experts involved in secondary support were attending physicians (*n* = 80, 28.6%), followed by psychologists (*n* = 58, 20.7%) and child life specialists (*n* = 36, 12.9%).

**Table 2:** Checked items and experts who supported the needs of adolescent and young adult cancer patients (AYAs)

Checked items											
	*N*	%		*N*	%		*N*	%		*N*	%
Physical problems			Family problems			Practical problems			Emotional problems		
Total (*n* = 361)	244	67.6	Total (*n* = 361)	91	25.2	Total (*n* = 361)	196	54.3	Total (*n* = 361)	167	46.3
Inpatients (*n* = 304)	202	66.4	Inpatients (*n* = 304)	67	22.0	Inpatients (*n* = 304)	149	49.0	Inpatients (*n* = 304)	122	40.1
Outpatients (*n* = 57)	42	73.7	Outpatients (*n* = 57)	24	42.1	Outpatients (*n* = 57)	47	82.5	Outpatients (*n* = 57)	45	78.9
Pain	105	29.1	Physical or mental health of family members	54	15.0	Work or school	130	36.0	Anxiety	123	34.1
Fatigue	88	24.4	Dealing with partner	33	9.1	Information about illness or treatment	94	26.0	Worry	105	29.1
Appearance	75	20.8	Interaction with parents	32	8.9	Money	85	23.5	Depression	86	23.8
Eating	69	19.1	Dealing with children	29	8.0	Treatment options	74	20.5	Fear	86	23.8
Sleep	66	18.3	Interaction with other family members	13	3.6	Quality of life during hospitalization	56	15.5	Sadness	60	16.6
Daily activity	58	16.1				Ability to have children	38	10.5	Nervousness	59	16.3
Dryness or itchiness of skin	45	12.5				Transportation	36	10.0	Loss of interest in usual activities	27	7.5
Constipation	44	12.2				Important schedule or events	32	8.9	Irritation	26	7.2
Tingling of hands or feet	44	12.2				Interaction with people other than your family	30	8.3	Confusion	7	1.9
Nausea	38	10.5				Someone to talk to or consultation environment	27	7.5	Emotional ups and downs	3	0.8
Bathing or dressing	35	9.7				Childcare	25	6.9	Loneliness	1	0.3
Breathing	34	9.4				Interaction with medical staff	20	5.5	Anger	1	0.3
Nasal dryness or congestion	31	8.6				Housing	20	5.5			
Indigestion	26	7.2				Sexual issues	17	4.7			
Changes in urination	25	6.9				Heredity	7	1.9			
Feeling swollen	25	6.9				Hobby and exercise	6	1.7			
Diarrhea	22	6.1				Available systems and services	6	1.7			
Fever	22	6.1				Love and marriage	4	1.1			
Memory or concentration	20	5.5				Eating habits	3	0.8			
Consultation with experts	9	2.5				Not being proactive	1	0.3			
Mouth sores	8	2.2				Spiritual or religious concerns	1	0.3			
Use of non-prescription medicine	5	1.4				Not respected	0	0.0			
Itching	0	0.0				Not understood	0	0.0			

Experts who supported the needs of AYAs												
	Total (*n* = 280)		Inpatients (*n* = 256)		Outpatients (*n* = 24)		Cancer center (*n* = 196)		General hospital (*n* = 77)		Pediatric hospital (*n* = 7)	
	*N*	%	*N*	%	*N*	%	*N*	%	*N*	%	*N*	%
Attending physician	80	28.6	77	30.1	3	12.5	75	38.3	4	5.2	1	14.3
Psychologist	58	20.7	50	19.5	8	33.3	10	5.1	44	57.1	4	57.1
Child life specialist	36	12.9	36	14.1	0	0.0	36	18.4	0	0.0	0	0.0
Pharmacist	23	8.2	23	9.0	0	0.0	19	9.7	2	2.6	2	28.6
Rehabilitation staff	21	7.5	21	8.2	0	0.0	15	7.7	6	7.8	0	0.0
Specialized nurse	16	5.7	8	3.1	8	33.3	6	3.1	10	13.0	0	0.0
Medical social worker	13	4.6	11	4.3	2	8.3	12	6.1	1	1.3	0	0.0
Palliative care team	10	3.6	10	3.9	0	0.0	6	3.1	4	5.2	0	0.0
Psychiatrist	9	3.2	9	3.5	0	0.0	9	4.6	0	0.0	0	0.0
Nutritionist	3	1.1	3	1.2	0	0.0	3	1.5	0	0.0	0	0.0
Appearance care staff	3	1.1	3	1.2	0	0.0	3	1.5	0	0.0	0	0.0
Child support	2	0.7	0	0.0	2	8.3	0	0.0	2	2.6	0	0.0
Others	6	2.1	5	2.0	1	4.2	2	1.0	4	5.2	0	0.0

### The extent to which the program was implemented as intended (Implementation)

Of the 8 institutions, 5 targeted inpatients, 2 targeted outpatients, and 1 targeted both outpatients and inpatients. The implementation status and the characteristics of each institution are shown in Table 3.

**Table 3:** The implementation status and characteristics of each institution

Institutions		Inpatients or outpatients	Occupation in charge	Targeted department or ward	AYAs^a^ at each institution during eligibility period^b^	AYAs^a^ in the targeted department or ward^b^	First screening^b^		Second screening^b^	
					*N*	*N*	*N*	%	*N*	%
Cancer Center	National Cancer Center Hospital	Inpatients	Nurse	Breast and medical oncology, gynecology	17,637	243	218	89.7	128	52.7
	Shizuoka Cancer Center	Inpatients	Nurse	Pediatrics, orthopedics, adolescent and young adult ward	1771					
	Aichi Cancer Center	Outpatients	Nurse	Hematology, thoracic surgery, gastrointestinal surgery, orthopedics, breast	1298					
General Hospital	Nagoya City University Hospital	Inpatients	PCT^c^	Orthopedics, gynecology, urology, PCT^c^	9540	100	90	90	44	44
	Nagoya Medical Center	Inpatients	Nurse	Pediatrics, hematology, medical oncology, breast, urology, gynecology	2790					
	St. Luke’s International Hospital	Outpatients	Nurse	Breast surgery	974					
Pediatric hospital	Saitama Prefectural Children’s Medical Center	Inpatients or outpatients	Nurse, attending physician	Hematology and oncology	34	18	18	100	8	44.4
	National Center for Child Health and Development	Inpatients	Liaison doctor	Pediatric cancer center	707					
Total					34,751	361	326	90.3	180	49.9

^a^Adolescent and young adult cancer patients

^b^Including duplicates

^c^Palliative care team

The percentage of participants who underwent the first screening was 90.3% (326/361), which was higher than the 90% we had defined as the cutoff indicating feasibility. The interviews identified the following factors in cases where screening could not be provided: (a) patients were in poor general condition due to emergency hospitalization; (b) patients refused to respond to a request for screening because they were repeatedly screened each time they were admitted to the hospital for a short period of time; and (c) nurses were too busy.

### How well the program worked (Effectiveness)

Both the DT score and the number of checked items on the problem list were significantly lower after the program, both among all patients (including those who missed the second screening; *p* < 0.05) and among those who completed both the first and second screenings (*p* < 0.05) (Table 4). There was a mean of 18.6 days (SD 17) between the first and second screenings. The rates of AYAs with high distress were significantly lower after receiving the support program (37 of 166, 22.3%) compared with before (108 of 322, 33.5%) (*p* < 0.05).

**Table 4:** Score differences before and after the support program and days between first and second screenings

Score difference before and after the support program (*t*-test, 2-sided test)							
	Pre		Post				
	*M*	*SD*	*M*	*SD*	*t*	*p*	*Cohen’s d*
Total (including the number who missed the second screening) (*n* = 326)							
Distress Thermometer	3.4	2.9	2.8	2.3	20.8	< .001	0.21
Number of checked items on problem list	6.5	7.3	2.4	4.0	16.9	< .001	0.71
Only those who underwent both first and second screenings (n = 180)							
Distress Thermometer	3.5	2.9	2.8	2.4	3.7	< .001	0.27
Number of checked items on problem list	7.7	6.2	5.0	4.5	6.9	< .001	0.50
Days between first and second screenings							
	*M*	*SD*	*Min*	*Max*			
Inpatients and outpatients (*n* = 180)	18.6	17.2	1	90			
Inpatients (*n* = 130)	14.6	16.4	1	90			
Outpatients (*n* = 50)	29.0	14.6	7	57			

### The sustainability of the program as part of routine care (Maintenance)

After this study ended, the program was continued at all 8 participating institutions as part of routine care. Reported factors that increased sustainability were as follows: (a) understanding the differences in support and resources between institutions; (b) increased awareness by healthcare providers that AYAs require support; and (c) establishment of a consultation service to support AYAs, thus facilitating medical treatment and multidisciplinary cooperation. Reported barriers to sustainability were as follows: (a) an increased daily burden in clinical practice; (b) challenges in gaining an understanding of the entire institution (including other departments) so as to maintain the system; and (c) ensuring high-quality support for identified needs.

## Discussion

In this multicenter, retrospective study, we used the RE-AIM to demonstrate the feasibility and preliminary effectiveness of a psychosocial support program based on the DTPL-J for AYAs.

### Program setup (Adoption)

AYAs want to receive a controllable amount of information from their healthcare professionals and to participate in decision-making about their care [34]. The factors that facilitated setup in this study were consistent with those in the previous report [34]. Furthermore, the barriers to setup in this study were an increased burden on both patients and healthcare workers and the need to gain the approval of related departments. These findings were consistent with those of a previous study identifying person-centered factors, service-related factors, and systemic factors as hindrances [35].

### Program participants (Reach)

The participation rate was 90.3%, which was higher than the 90% that we defined as the cutoff for feasibility. Since most of the subjects (84.2%) were inpatients, this program may be more easily implemented in this population. Although outpatients were more likely than inpatients to check items in all 5 categories of the problem list, the number of the types of experts who provided support was higher for inpatients (12 types) than outpatients (6 types). Future research should address the implementation of this program in outpatient settings.

Patients who participated in the program included many patients with cancer types with high support needs. In bone or soft tissue cancer, the risk of chemotherapy affecting fertility has been reported [36, 37]. Breast cancer patients were reported to have psychological distress, especially fear of cancer recurrence [38]. In gynecologic cancer, cancer has a direct effect on fertility. In hematological cancer patients, especially in patients who undergo hematopoietic stem cell transplantation, they often endure much longer and consequently more disruptive treatment interventions which may result in a greater likelihood of side effects and late effects. Many AYAs who undergo hematopoietic stem cell transplantation reported to have described unmet psychological needs [39]. Brain tumor patients were reported to have emotional and physical distress encompassing: fatigue, fears, memory and concentration, and worry [40].

The checked items in the problem list mostly concerned practical and emotional issues. The top 5 were work problems or school problems (*n* = 130, 36.0%), anxiety problems (*n* = 123, 34.1%), worry problems (*n* = 105, 29.1%), pain problems (*n* = 105, 29.1%), and fatigue problems (*n* = 88, 24.4%). These problems are consistent with previous studies in AYAs in Asia [21] as well as in Australia, the UK, and the USA [18]. AYAs may commonly experience a wide range of physical symptoms and psychological and social problems regardless of race or ethnicity.

Attending physicians most commonly provided inpatient support, while specialized nurses and psychologists were most likely to care for outpatients. This discrepancy may be related to differences between inpatients and outpatients in terms of their access to attending physicians. In Japan, support systems and activities for AYAs vary widely across hospitals, and both consultations for unmet needs and the provision of information remain insufficient [41]. Therefore, it is expected that this program will be disseminated and implemented in other locations, as suggested by the fact that multidisciplinary experts may be involved depending on the resources at each institution.

### The extent to which the program was implemented as intended (Implementation)

The program was mainly used in departments that treat cancer types that frequently affect AYAs, such as breast oncology, orthopedics (bone and soft tissue oncology), hematological oncology, and pediatrics. This suggests that research cooperation was easily obtained from departments with a high need for AYA support [42].

Screening could not be provided in cases of emergency hospitalization or refusal to repeat screening in a short period of time. The appropriate timing and frequency of screening should be considered. Another challenge was how to efficiently provide screening in a busy clinical practice.

There was a need for this program among healthcare workers; however, it could be burdensome for both patients and healthcare workers. Future studies should consider ways to provide support programs that are less burdensome for all involved.

### How well the program worked (Effectiveness)

The DT score, the number of checked items on the problem list, and the rates of AYAs with high distress were significantly lower after this program based on the DTPL-J for AYAs, suggesting its preliminary effectiveness. In a previous study, DT scores decreased significantly over time [21]. However, while the evaluation interval in that study was 6 months, it was only 18.6 days in this study. The fact that the number of checked items decreased in this study suggests that because multidisciplinary experts were involved according to each patient’s needs, the program helped decrease DT scores, the number of checked items, and the rates of AYAs with high distress.

Though the rate of AYAs with high distress before receiving the support program was 33.5%, a previous study of AYAs within 3 months of diagnosis reported that 42% experienced distress using the same cutoff (DT of 5) of this study [18]. Our study might have included AYAs who were diagnosed long ago because the duration after diagnosis was not included in the eligibility criteria. The prevalence of distress in this study might be low compared to the prevalence in the previous study because the distress of AYAs decreases as time after diagnosis increases [21]. In a previous study using a different cutoff value (a DT score of ≥ 4 points) from that in the present study, the rate of AYAs with high distress decreased by 15.4% over a 6-month time course after cancer diagnosis [21]. The 11.2% reduction in AYAs with high distress over the only 18.6 days interval in this study suggested the preliminary effectiveness of this program.

### The sustainability of the program as part of routine care (Maintenance)

After this study ended, the program was continued at all 8 institutions as part of routine care because of its benefits regarding the increased understanding of and the need for AYA support. However, there are challenges concerning the burden on both patients and providers and the need to gain an understanding of the entire institution. Just as psychosocial support for AYAs is a national project in Australia [23], it needs to be addressed as a national project in Japan in order to expand and maintain the program beyond the eight institutions in this study. The content of the program needs to be improved, and evidence needs to be gathered to lead to appropriate medical fee additions to the program.

This study suggests the feasibility and preliminary effectiveness of a psychosocial support program based on the DTPL-J for AYAs. The program allows healthcare workers to identify distress and determine the supportive care needs of AYAs, and to address these needs.

A previous study showed that AYAs in Japan had a threefold higher risk of major depressive disorder within 6 months before and 12 months after cancer diagnosis compared with cancer‐free controls [43]. Furthermore, another study revealed that among AYAs aged 15–24 years, the risk of suicide was elevated compared with cancer‐free controls [44].

Our findings suggest that the program using the DTPL-J for AYAs may lead to cross-specialty collaboration and improved performance of multidisciplinary teams that support AYAs. This may lead to early palliative care for patients with cancer [45]. As a next step, we will determine if this program can prevent psychiatric disorders and suicides in AYAs. Although support for AYAs is being promoted as a nationwide policy in Japan, it has not been implemented clinically. The use of this program to support AYAs may help support this population in practice.

This study has several limitations. First, it used a retrospective, observational design that may have caused several systematic biases. The effectiveness of the program for addressing AYAs’ distress and needs may have been overestimated because of bias, for instance, due to the effect of other factors such as the support of family members. To evaluate the effectiveness of the program, a prospective trial is needed as a next step. Second, the 8 participating institutions all belonged to a research group defined by a Grant-in-Aid, raising the question of institutional bias. Although the median number of newly diagnosed AYAs per hospital is only 3 per year in Japan [35], the number of AYA registrations at the 8 institutions was quite large because at least 34 patients including duplicates were seen at each institution during the 8-month eligibility period. Thus, the results of this study may not be applicable to other settings, with the exception of comprehensive cancer centers and teaching hospitals. Third, the diagnosis and treatment level of each institution in different regions and the support provided by multidisciplinary team members to patients are different. Though an implementation manual included the role of each multidisciplinary expert and how they should support AYAs in order to ensure the professionalism of psychosocial support, this study allowed for adaptation to the actual conditions at each institution to increase clinical feasibility. Fourth, since the number of targeted AYAs at each institution comprised only a small proportion of the total number of AYAs, careful interpretation is required. Finally, the subjects of this study were all Japanese. This should be taken into account when applying our methodology to other races and ethnic groups.

## Conclusions

This study suggests the feasibility and preliminary effectiveness of a psychosocial support program based on the DTPL-J for AYAs.

## Data Availability

The data that support the findings of this study are available from the corresponding author upon reasonable request.
